# Why can’t they “Go back”? — A multi-group comparative analysis of pre-service teachers’ hometown return intention in local universities based on latent profile analysis

**DOI:** 10.1371/journal.pone.0360231

**Published:** 2026-10-15

**Authors:** Chen Qing, Yu Zhang, Zhichao Wang, Haiying Yu

**Affiliations:** 1 Faculty of Education, Northeast Normal University, Changchun, Jilin, China; 2 Academy of Educational Sciences, Mudanjiang Normal University, Mudanjiang, Heilongjiang, China; Pingdingshan University, CHINA

## Abstract

The high-quality development of rural education urgently requires strengthening the supply of outstanding rural teachers. Based on Social Cognitive Career Theory (SCCT), a survey of 6,546 teacher education students from local universities in Heilongjiang Province revealed that the willingness to teach in rural areas among three identified profiles—the Weak Foundation Profile, the Development-Oriented Profile, and the Excellence-Prepared Profile—is significantly influenced by academic year and family capital. This influence manifests differentiated patterns via the “environment-person-behaviour” transmission pathway: family support acts as a common motivating factor for rural teaching willingness across all three groups; for foundation-weak students, professional competence primarily exerts a mediating effect through the family support pathway; for development-oriented students, professional competence shows no significant mediating effect in this pathway; and for excellence-oriented students, the willingness to return to their hometowns for rural teaching is jointly driven by the dynamic interplay between environmental factors and individual professional competence. Therefore, a differentiated approach is imperative: policy orientation should strengthen the enabling role of family support, optimise the pathways for localised training and incentives for rural teachers, and establish a growth system for rural education leaders. These measures will further refine the long-term mechanisms for attracting and retaining talent in rural teaching positions.

## 1. Introduction

The development of rural education hinges on teachers, and the cultivation of the rural teaching workforce must be accorded strategic priority. Ensuring that high-calibre rural teachers are willing to take up posts in rural areas, able to remain there, and capable of delivering effective teaching constitutes a fundamental prerequisite for improving educational quality and realising the full potential of education. From the perspective of the “rational economic man”, the distance between their place of birth and the institution is a key factor influencing teacher trainees’ decision to take up rural teaching posts [1], Establishing a cadre of “local” teachers to encourage trainee teachers to take up posts in rural areas represents an effective approach to reducing career transition costs and enhancing the stability of their rural teaching commitments. As a vital reserve force for rural teaching staff, whether graduates from local universities return to teach in their hometowns directly impacts the quality and structural stability of rural teacher recruitment. More profoundly, it influences the effectiveness of addressing the imbalance in educational resources between urban and rural areas, serving as a crucial link between “high-quality rural education development” and “building a high-calibre rural teaching workforce”.

To cultivate a dedicated, high-calibre, and dynamic rural teaching workforce, China has progressively deepened targeted rural teacher training policies since the 21st century. These include the Rural Teacher Support Programme (2015–2020), the New Era Basic Education Teacher Strengthening Plan, and the Special Post Programme—which has recruited 1.18 million teachers over two decades. These progressive policies have provided robust institutional support and resource guarantees for supplying high-calibre teaching staff to rural areas. Nevertheless, the actual outcomes of these policies remain significantly below expectations. Constrained by practical challenges — recruiting talent is difficult and retaining it is even harder — some regions have seen the emergence of teachers with urban-rural dual-residence patterns. Moreover, the overall willingness among post-2000s normal school graduates to teach in rural areas remains relatively low [2]; the phenomenon of targeted teacher training students presents a paradox where low-quality compliance coexists with high-quality defiance [3], this has heightened the risk of substandard teacher supply in rural areas.

The supply efficiency and retention quality of rural teachers are pivotal to the high-quality development of rural education. The deployment of high-calibre teaching staff to rural areas and their stable retention constitute essential prerequisites for enhancing the professional competence of rural school teaching teams and optimising the structure of the rural teaching workforce. Exploring and analysing the key factors influencing the willingness of local university teacher education students to teach in rural areas is a necessary prerequisite and vital foundation for addressing the aforementioned issues.

Academic research into factors influencing teacher education students’ rural teaching engagements generally unfolds across three dimensions. Firstly, from a motivational perspective, studies examine the external forces and intrinsic motivations driving teacher education students towards rural teaching. Among these external motivators are the level of higher education institutions and the “localised” value orientation within university teacher training programmes [4], Local government policy support [5], Internal motivational factors include pre-service teachers’ perceptions and attitudes towards the teaching profession [6], Career Development Plan [7], Employment Awareness [8], Understanding and integration into rural life [9], Policy awareness [10]. Moreover, the personal characteristics of teacher training students—such as gender, age, subject specialisation, year group, personality, and parents’ educational attainment—have also been extensively discussed in relation to their willingness to teach in rural areas.

Secondly, from the perspective of teachers’ career development, this research examines the key factors influencing pre-service teacher candidates’ commitment to rural teaching during different stages, such as the pre-service training period and the post-service novice teacher development phase. During the pre-service teacher education stage, pre-service preparation and training play a crucial role in shaping their professional cognition and strengthening their commitment to rural teaching [11], theoretical study and educational practice [12] helps teacher training students strengthen their resolve to pursue a career in teaching; improvements in academic performance and teaching skills can enhance their confidence in rural teaching [13]; teacher training students’ teaching competence directly shapes the development of their professional interest in the teaching profession [14]; the theoretical framework of “Factors Influencing Teaching Choice”, developed by Watt and Richardson, indicates that pre-service teachers’ professional endeavour planning, professional development expectations, career persistence planning, and leadership position aspirations significantly influence their career orientation [15]. During the post-employment development phase of novice teachers, factors such as social role models, pre-existing social connections, salary levels, and housing support arrangements significantly influence the job stability of teacher training graduates who take up rural teaching positions [16,17]; social status, cross-regional teaching appointments, and professional training quality will influence the formation of their professional identity as rural teachers [18].

Thirdly, from a typological perspective, this study examines differences in hometown teaching intentions and career choices across different profiles of teacher education students. In terms of typological classification, for instance, we can categorise teacher education students based on their career inclinations [19], classified as proactive and enterprising, proactive and dedicated, or passive and subsistence-oriented, these groups are distinguished on the basis of professional identity [20], classified into the passionate type, the interest-driven type, the utilitarian type, and the avoidance type; based on the willingness to relocate [21], classified into stable and unstable types; Additionally, based on willingness to teach [22], classified into protocol type, free type, and premium type. The above indicates that although academic circles have conducted extensive research on the factors influencing pre-service teachers’ commitment to rural teaching, empirical studies on how the quality of pre-service training at local universities affects their willingness to teach in rural areas remain relatively scarce. Most studies exploring the formation mechanisms underlying such willingness have focused on the perspectives of pre-service teachers’ career inclination and professional identity.

Professional competence, defined as the comprehensive qualities exhibited by practitioners in their professional activities, refers to the work ethics and behavioural norms cultivated by practitioners in accordance with the inherent requirements and standards of their profession. The professional competence of teacher education students in local universities represents a critical capacity to meet the developmental needs of rural education and support their lifelong professional growth as educators [23], it is of great significance for these prospective teachers’ future performance in rural teaching and their sustainable career development, while also serving as a key benchmark for evaluating the quality of their pre-service training.

As individuals mature, learning motivation, interests, opportunities, and resources exert an increasingly strong influence on their development [24], the distinctiveness of teacher education students’ learning experiences and their individual heterogeneity are becoming increasingly pronounced. Significant individual differences in their perceptions of the rural teaching profession, developmental expectations, and policy awareness inevitably lead to typological differentiation in the development of their professional competence.

Among existing typological studies, few have specifically classified pre-service teachers’ readiness for rural teaching according to their developmental stages of professional competence during their pre-service training. Analysing the heterogeneity in pre-service teachers’ professional competence and comparing the mechanisms underlying their hometown return willingness can provide implications for optimising pre-service training for local rural teachers and improving the retention of graduates from local universities in rural schools.

Social Cognitive Career Theory (SCCT) is a representative theory in the field of career development [25], proposed by Lent, Brown and Hackett in 1994. This theory divides the core drivers of individual career decision making into three core parts including external environmental conditions, personal cognitive traits and individual behavioral tendencies. This theory regards professional self-efficacy, occupational competence, and career cognition as pivotal factors linking environmental influences to career intentions. It emphasises that individuals’ assessments of their own professional capabilities and perceptions of occupational value serve as crucial bridges that transform environmental factors into career choices. That is, individuals develop preferences for specific occupations under the combined influence of environmental shaping and personal factors. Based on this theory, the willingness of teacher education students at local universities to teach in rural areas is the result of the interplay between external environmental factors and individual student characteristics, shaped through the “environment–person–behaviour” transmission pathway.

Academic research indicates that family support, institutional training, external policies, and social environments profoundly influence teacher education students’ willingness to teach in rural areas. Integrating these factors with professional competence development—the key individual growth factor during their pre-service training—enables a typological investigation into the mechanisms shaping this willingness. This approach provides a pathway for optimising the localised training and supply system for rural teachers. This study draws upon the Social Cognitive Career Theory (SCCT) proposed by Lent, Brown and Hackett, focusing on the core question of why pre-service teachers from local universities are reluctant to teach in rural areas.

The theoretical logic of this study fully aligns with the core framework of SCCT as reflected in the research model presented in Fig 1. On the contextual level, the model contains three groups of environmental variables which serve as external supports and constraints affecting students’ career decision making. They mainly involve family environment, university environment and macroeconomic policy environment. Such objective situational conditions help form pre-service teachers’ overall views on rural teaching work. On the individual level, the model covers multiple personal variables that match the cognitive related mechanisms emphasized by SCCT. These elements include professional learning literacy, practical teaching competency, occupational post adaptability, career development planning and rural educational perception. All these individual indicators can effectively reflect pre-service teachers’ professional self efficacy, outcome expectation and inner recognition of educational occupations. Following the basic path of SCCT, relevant environmental conditions and individual inner conditions will together affect the final research outcome reflected by career choice tendencies. This part mainly refers to students’ willingness to integrate into therural environment, their willingness to teach at rural schools and their willingness to interact with rural teachers. This progression from contextual factors to individual cognition and competence and finally to career intention underpins the entire research design. This study therefore focuses on these three layers of factors to examine how they jointly shape pre-service teachers’ willingness to teach in rural areas (Fig 1).

**Fig 1:**
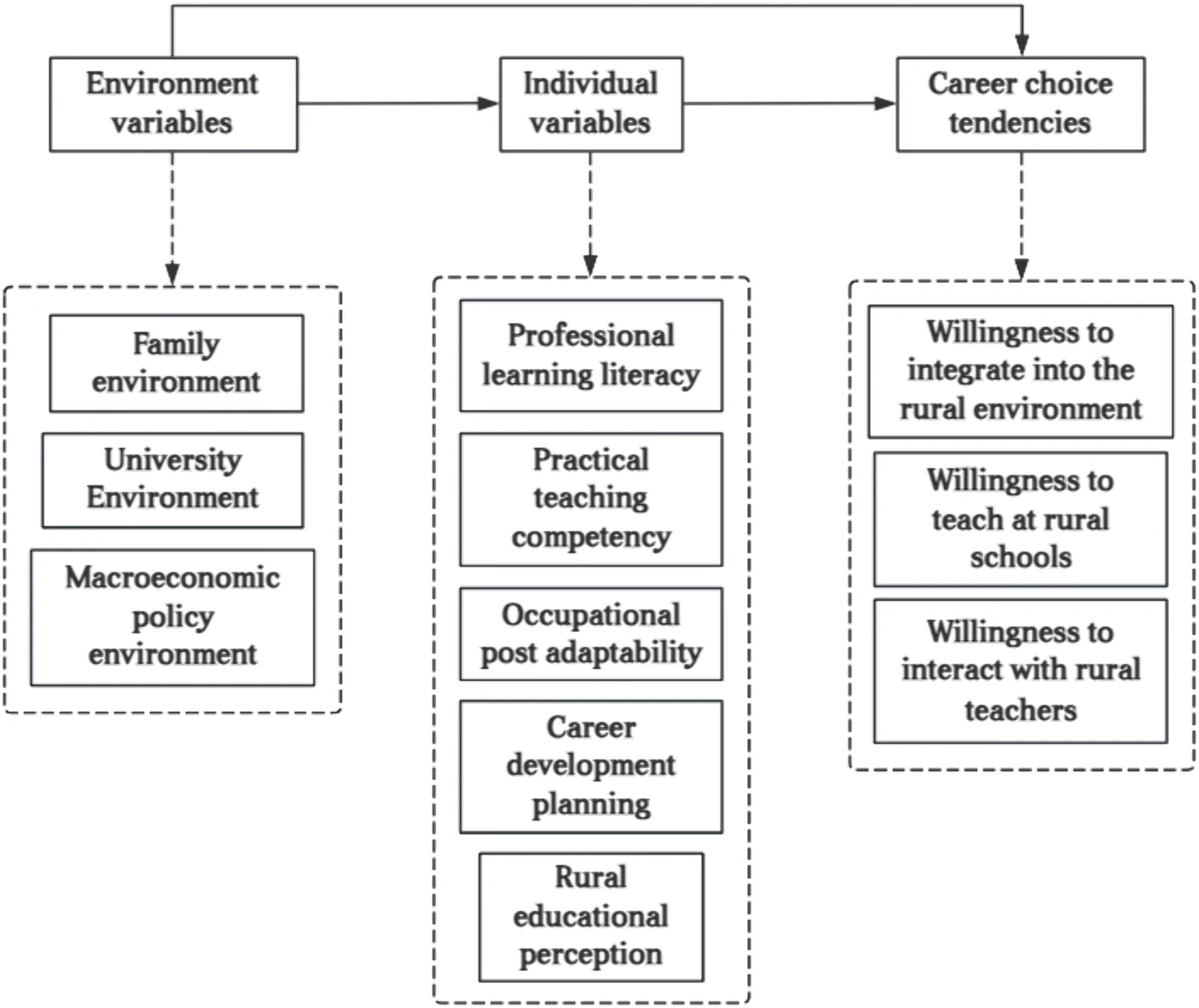
Research theoretical model of career choice tendencies among local university pre-service teachers.

Given the internal heterogeneity in the professional competence of teacher education students at local universities, this study employs Latent Profile Analysis (LPA) to delineate the typological characteristics of student cohorts at different developmental stages of professional competence. LPA is an individual-centred statistical method that determines the optimal classification based on participants’ response patterns to manifest variables, and it uses objective model fit indices to maximise the accuracy and reliability of classification outcomes [26], this thus serves as a suitable tool for analysing group differences in the professional competence of pre-service teachers.

Building on Latent Profile Analysis (LPA) results, this study adopts multi-group comparison to examine differences in pre-service teachers’ willingness to return to their hometowns for teaching across distinct professional competence profiles, as well as the underlying mechanisms driving these differences.Different from previous studies that mostly regard pre-service teachers as a single homogeneous group for unified analysis, this study employs latent profile analysis to fully reveal the heterogeneous developmental characteristics of pre-service teachers’ professional competence and its differentiated impact on rural teaching willingness. Furthermore, multi-group comparison strategy can further distinguish the heterogeneous action paths of external environmental factors and individual inherent factors among different groups of pre-service teachers, which deepens the hierarchical and refined understanding of rural teacher supply dilemma. These rigorous empirical research designs not only enhance the overall academic rationality and research depth of this paper, but also form obvious research differentiation advantages compared with existing relevant literatures, and lay a solid theoretical and empirical foundation for putting forward targeted and differentiated teacher training optimization schemes. The findings of this study are intended to propose actionable optimisation strategies, thereby providing further empirical support for targeted guidance programmes that support pre-service teachers from local universities in returning to their hometowns to take up rural teaching positions.

## 2. Research design

### 2.1. Research data

This study employed a stratified multi-stage sampling method. The participants were students majoring in teacher education from local undergraduate universities in 3 prefecture-level cities in Heilongjiang Province, China. Informed consent was obtained from participants prior to participation, and a total of 6,800 questionnaires were distributed.

Data screening and cleaning were conducted to eliminate invalid responses, including incomplete questionnaires, straight-lined responses, logical inconsistencies, and outliers. Cases with more than 10% item-level missing responses were directly excluded. For the remaining observations with limited missing data (<5%), listwise deletion was adopted for analysis. After data processing, 6,546 valid questionnaires were retained, yielding a valid response rate of 96.26%.

In terms of gender distribution, the sample comprised 1,196 males (18.27%) and 5,350 females (81.73%). Regarding academic year distribution: 2,258 first-year students (34.49%), 1,489 second-year students (22.75%), 1,515 third-year students (23.14%), and 1,284 fourth-year students (19.62%). By place of household residence: 3,206 students (48.98%) were from cities and counties, 1,547 students (23.63%) from townships, and 1,793 students (27.39%) from villages. By only child status: 3,610 students (55.15%) were only children, and 2,936 students (44.85%) were non-only children. Regarding household annual income distribution: 3,840 students (58.66%) had an income of RMB 50,000 or below; 1,681 students (25.68%) had an income between RMB 50,000 and RMB 100,000; 670 students (10.24%) had an income between RMB 100,000 and RMB 150,000; and 355 students (5.42%) had an income above RMB 150,000.

### 2.2. Measurements

This study developed and validated two questionnaires based on a systematic review of literature on teacher professional competence and rural teaching willingness, combined with theoretical frameworks, policy documents, and the practical context of rural teacher training in Heilongjiang Province, China. The initial dimension system was constructed based on classic scales and mainstream theories, and an item pool was formed through open interviews, expert consultation, and literature induction. An initial item pool was generated, followed by two rounds of expert validity assessment with five specialists in teacher education and educational psychology. Experts were invited to evaluate the relevance, clarity, representativeness, and accuracy of each item; items with a content validity index (CVI) < 0.80 were deleted or revised. A pilot test with 280 teacher education students was then conducted to refine item wording, eliminate ambiguous items, and improve readability. Item analysis (critical ratio method, correlation analysis) and exploratory factor analysis (EFA) were carried out to delete unqualified items, ensuring each item had good discrimination and structural attribution. Using this process, the final questionnaires with stable structure were finalized for formal investigation.

The first questionnaire measures professional competence among teacher education students and covers five dimensions: professional learning literacy, practical teaching competency, occupational post adaptability, career development planning, and rural educational perception. Specifically: professional learning literacy includes the academic performance in professional courses, practical professional skills, and the innovative application of professional knowledge, which comprehensively reflects students’ mastery and flexible application of disciplinary professional knowledge during their university studies; practical teaching competency covers teaching proficiency, classroom management capabilities, and instructional design and curriculum implementation abilities, focusing on students’ comprehensive practical capabilities required for front-line teaching work; occupational post adaptability involves students’ preparation for teaching roles, self-confidence in teaching in rural schools, progress in obtaining teaching qualifications, and emergency response and problem-solving abilities in teaching scenarios, which reflects the overall readiness of pre-service teachers to engage in educational and teaching work; career development planning reflects pre-service teachers’ visions and systematic designs for future professional growth paths, industry positioning, and long-term educational career development; rural educational perception involves pre-service teachers’ objective understanding of the operating mechanism and educational resources of rural educational institutions, their adaptability to rural living and working environments, and their cognitive recognition of the development value and practical significance of rural basic education.

The questionnaire employs a five-point Likert scale, ranging from “1” (strongly disagree) to “5” (strongly agree), with higher scores indicating better professional competence development among local university teacher education students. The overall Cronbach’s alpha coefficient of the questionnaire was 0.897, far higher than the critical value of 0.70, indicating excellent internal consistency reliability. Confirmatory factor analysis (CFA) was used to verify the construct validity of the five-factor model. The results showed that χ²/df = 7.943, RMSEA = 0.089, TLI = 0.986, CFI = 0.995, and GFI = 0.986. According to commonly accepted fitting criteria: χ²/df < 8 is acceptable; RMSEA < 0.09 is reasonable for large-sample research; TLI, CFI, and GFI all above 0.90 indicate good model fit. Although RMSEA is slightly higher than 0.08, this phenomenon is common in large-sample studies due to increased sensitivity of the chi-square statistic. Overall, the model presents satisfactory construct validity.

The questionnaire on factors influencing rural teaching willingness among teacher education students at local universities comprises two sections. The first section examines the environmental factors affecting their willingness to teach in rural areas, focusing on three dimensions: family support, university teacher training objectives, and policy orientation.

Academic research indicates that when individuals experience negative psychological perceptions towards rural teaching due to external influences, the inherent efficacy of internal support within the family organisation—as an ascribed characteristic—becomes particularly prominent [27], Parental opinions and household income profoundly influence an individual’s career planning; therefore, in the context of “family support”, three sub-dimensions—parental attitudes, parental expectations, and family economic situation—were selected to investigate the influence of the family micro-environmental system on the willingness of teacher training students from local universities to teach in rural areas.

As the primary training ground for rural teachers, the curriculum design and educational objectives of local universities exert a lasting impact on the knowledge, skills, and ideological behaviour of teacher training students. In particular, the teacher training programme is directly linked to the development of professional competence among trainee teachers [28], therefore, in the context of “university teacher training objectives”, five sub-dimensions were selected: regional talent cultivation positioning, localized curriculum system construction, contextualized practical teaching design, regionally embedded campus cultural education, and rural-oriented employment guidance mechanism. These dimensions were examined to investigate the influence of this meso-level institutional environment on the willingness of teacher training students at local universities to take up rural teaching posts.

From the perspective of the macro-environmental system, teacher trainees’ satisfaction with relevant policies significantly influences their motivation to enter the teaching profession, occupational identity [29] and professional efficacy [30], therefore, in the dimension of “policy orientation”, seven sub-dimensions were selected: special post policy guidance, salary and benefits improvement, preferential treatment in professional title evaluation and appointment, support for capacity building, incentives for professional identity, improvement of living conditions, and student loan compensation. These sub-dimensions were used to examine the macro-level factors influencing the willingness of local university teacher training students to teach in rural areas.

Part Two investigates the willingness of teacher education students at local universities to teach in rural areas. The questionnaire comprises three dimensions: willingness to integrate into the rural environment, willingness to teach at rural schools, and willingness to interact with rural teachers. A sample item is “Do you enjoy the environment of rural schools?”. The questionnaire employs a five-point Likert scale ranging from “1” (strongly disagree) to “5” (strongly agree). The Cronbach’s alpha coefficient was 0.884, indicating good internal consistency reliability. CFA was performed to test construct validity: χ²/df = 4.671, RMSEA = 0.085, TLI = 0.932, CFI = 0.981. All fit indices meet accepted criteria: χ²/df < 5, RMSEA < 0.09, and TLI & CFI > 0.90, demonstrating satisfactory construct validity.

### 2.3. Data analysis approach

This study employed Mplus 8.3 to conduct latent profile analysis, identifying developmental profiles of professional competence among teacher education students at local universities. Subsequently, descriptive statistics and chi-square tests were performed using SPSS 24.0 to compare the distributions of demographic variables across profiles, thereby examining the heterogeneous characteristics of rural teaching willingness among students at different professional competence development levels. Next, independent samples t-tests and one-way analysis of variance (ANOVA) in SPSS 24.0 were used to analyse variations in rural teaching willingness among teacher education students with different demographic characteristics across these developmental profiles. Finally, the study examined the generative mechanisms of rural teaching willingness among these students, with family support, university teacher training objectives, and policy orientation as independent variables, and professional competence development levels as the mediating variable.

## 3. Results

### 3.1. Descriptive statistics and correlation analysis

The mean values, standard deviations, and correlation coefficients for each variable in this study are presented in Table 1. The scores for the five components of professional competence development among teacher education students at local universities ranged between 3.39 and 3.80, indicating a moderate level. From highest to lowest, these were: practical teaching competency, professional learning literacy, occupational post adaptability, career development planning, and rural educational perception. The mean score for rural teaching willingness among local university teacher education students was 3.50, also indicating a moderate level. This suggests considerable scope for improvement in both the professional competence and rural teaching willingness of these students. Correlation analysis revealed significant positive correlations of low to moderate strength between variables (0.265 ≤ r ≤ 0.766, p < 0.01), with no high correlations observed. This pattern supports the feasibility of conducting cluster analysis.

**Table 1:** Mean, standard deviation, and correlation coefficient of each variable.

Variables	1	2	3	4	5	6
1.Professional learning literacy	1					
2.Practical teaching competency	0.766**	1				
3.Occupational post adaptability	0.582**	0.605**	1			
4.Career development planning	0.390**	0.388**	0.548**	1		
5.Rural educational perception	0.290**	0.304**	0.498**	0.408**	1	
6.Willingness to teach in rural areas	0.265**	0.289**	0.384**	0.266**	0.490**	1
Mean (M)	3.71	3.80	3.62	3.45	3.39	3.50
Standard Deviation (SD)	0.67	0.69	0.62	0.86	0.85	0.72

Note:*p < 0.05; **p < 0.01; N = 6,546.

### 3.2. Potential profile analysis

To investigate the latent categories of professional competence development levels among teacher education students at local universities, the number of profiles was incrementally increased. Model fit parameters were compared until the model with optimal fit was determined. Model fit evaluation metrics included: Log-Likelihood (LL), Akaike Information Criterion (AIC), Bayes Information Criterion (BIC), SSA-BIC, Entropy, Likelihood Ratio Test (LMR), and Bayes Likelihood Ratio Test (BLRT). Among these, smaller values of the model comparison metrics LL, AIC, BIC, and SSA-BIC indicate superior model fit. The Entropy metric, which is used to assess classification accuracy, is generally required to exceed 0.80, with values closer to 1 signifying greater accuracy. When the p-values of the likelihood ratio tests LMR and BLRT reach statistical significance (p < 0.05), it indicates that the k-category model is superior to the k-1 category model. Furthermore, subgroups accounting for less than 5% of the total sample were considered to lack substantive practical significance [31].

As shown in Table 2, fitting indices including LL, AIC, BIC and SSA-BIC declined with the rise in the number of profiles. The 4-profile and 5-profile solutions contained subgroups accounting for merely 0.7% and 0.8% of the overall sample respectively, such tiny proportions lacking practical research significance. Despite favorable entropy values across all models, the 3-profile model achieved an optimal balance of model fit, classification accuracy and theoretical rationality. Consequently, this study selected the three-profile solution as the optimal latent profile model, with subgroup proportions of 33.4%, 51.7% and 14.9% in sequence.

**Table 2:** Potential profile analysis model fit index.

*Model*	*Log likelihood*	*AIC*	*BIC*	*SSA-BIC*	*Entropy*	*LMR(p)*	*BLRT(p)*	*Percentage of sample*
1	−36113.44	72246.88	72314.75	72282.97	—	—	—	—
2	−32244.54	64521.08	64629.67	64578.83	0.771	<0.001	<0.001	0.483/0.517
**3**	**−29609.31**	**59262.63**	**59411.93**	**59342.02**	**0.944**	**<0.001**	**<0.001**	**0.334/0.517/0.149**
4	−28830.83	57717.66	57907.68	57818.70	0.958	<0.001	<0.001	0.007/0.503/0.335/0.155
5	−28262.08	56592.15	56822.90	56714.85	0.946	<0.001	<0.001	0.008/0.334/0.501/0.080/0.078

This study utilised the latent profile model indicators for the professional competence development levels of teacher education students at local universities to map the specific scoring patterns across three distinct profiles (Fig 2), alongside the standardised scores of the three professional competence profiles across different dimensions (Fig 3). Based on the varying levels of professional competence development among these teacher education students, the participant cohort was categorised into three groups: those with foundational weaknesses, those demonstrating developmental intent, and those exhibiting preparatory excellence.

**Fig 2:**
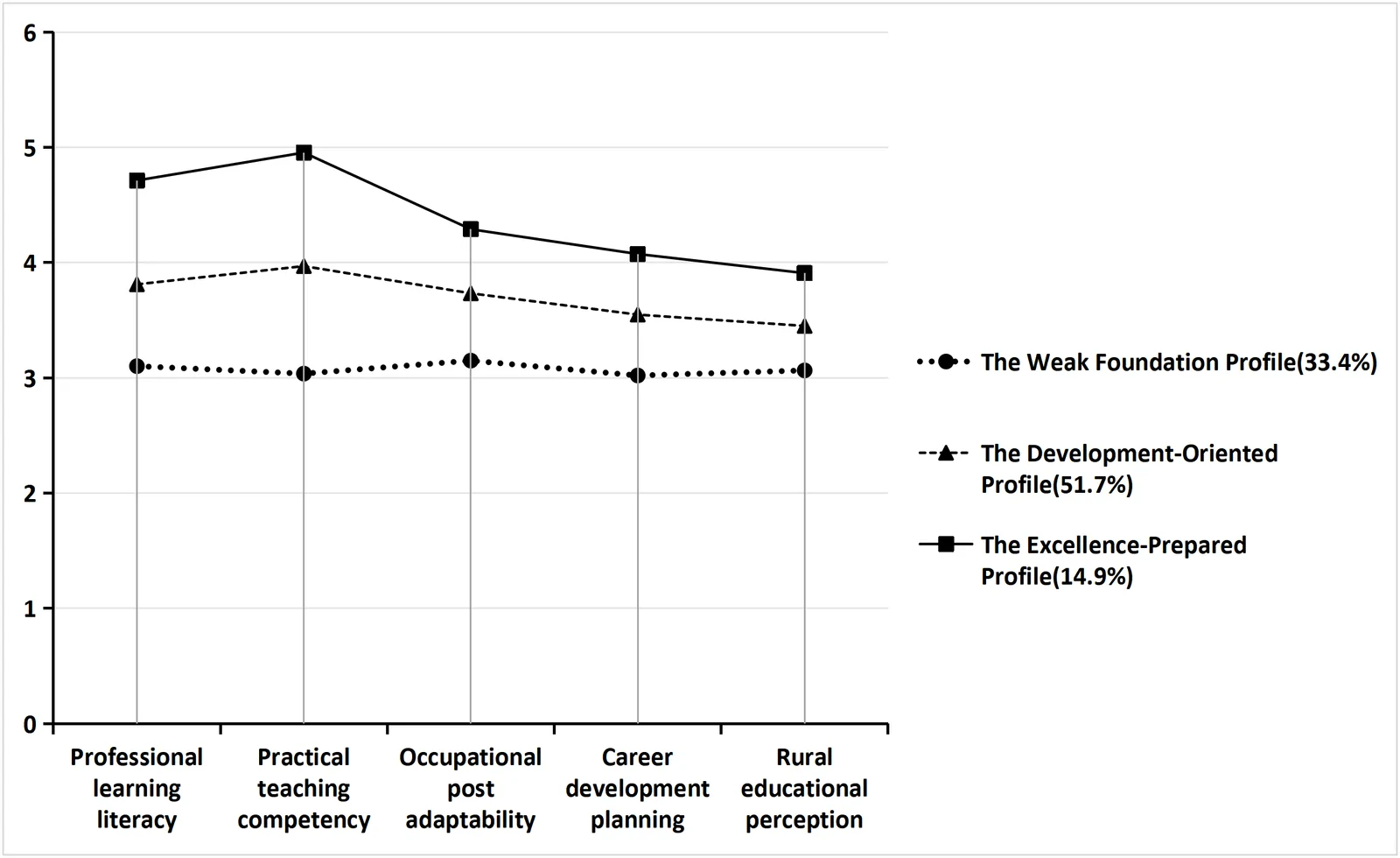
Potential profile of local university pre-service teachers across five core competency dimensions.

**Fig 3:**
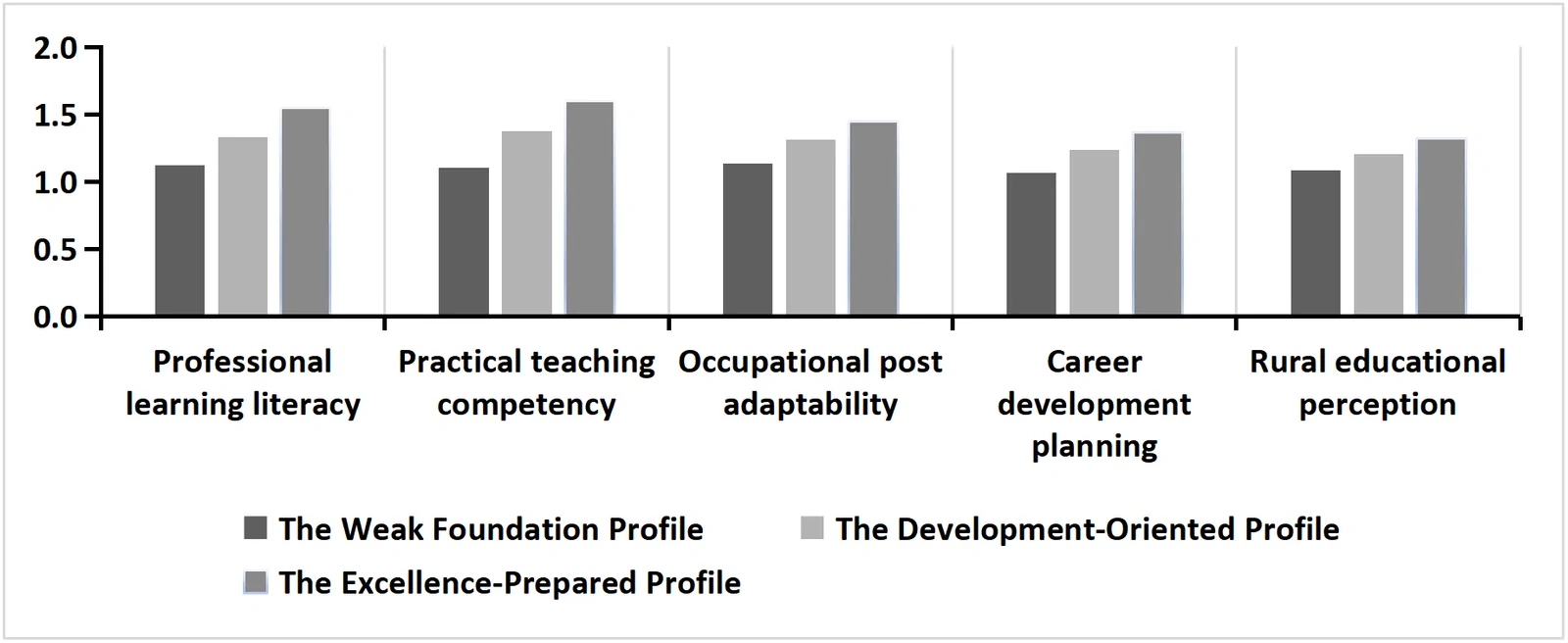
Standard scores of different profile results among local university pre-service teachers.

The first category comprises the Weak Foundation Profile of local university teacher education students (2188 individuals, accounting for 33.4%). This group scored the lowest across all five professional competence dimensions, exhibiting particular deficiencies in practical teaching competency, career development planning, and rural educational perception. They lack effective teaching strategies, demonstrate inadequate systematic planning for their future development, and possess a limited awareness of the grassroots educational environment in rural areas.

The second category comprises the Development-Oriented Profile of teacher education students from local universities (3,383 individuals, accounting for 51.7%). This cohort achieved moderate scores across all five dimensions of professional competence and constituted the largest group in the sample. They demonstrated solid subject knowledge, increasingly proficient teaching skills, a clear understanding of the teaching profession, and gradually crystallized career objectives. They possessed a sound grasp of the grassroots educational environment in rural schools, exhibited strong psychological adaptability, and showed considerable potential for development in rural teaching.

The third category comprises the Excellence-Prepared Profile of local university teacher education students (975 individuals, accounting for 14.9%). This group achieved the highest scores across all five dimensions of professional competence, demonstrating an outstanding level of development. They possess the characteristic of being “ready for immediate deployment” and represent high-potential talent for the development of the rural teaching workforce.

### 3.3. Heterogeneity analysis

Analysing demographic variations in the distribution of different professional competence development levels among teacher education students at local universities provides fundamental evidence for implementing tailored approaches in rural teacher training. Considering the identity characteristics of teacher education students at local universities, this study employs gender, year of study, hometown location, annual household income, and only-child status as independent variables. A chi-square test was conducted, with the professional competence category of these students as the dependent variable (Table 3). The findings indicate that distinct demographic variables exert a significant influence on the distribution of professional competence categories among teacher education students at local universities.

**Table 3:** Heterogeneity analysis.

Demographic Variables		N	The Weak Foundation Profile	The Development-Oriented Profile	The Excellence-Prepared Profile	X^2^	p
Gender	Male	1196	31.6%	45.4%	23.0%	77.054	<0.001
	Female	5350	33.8%	53.1%	13.1%		
Grade	Freshman (Year 1)	2258	31.0%	51.0%	18.0%	37.335	<0.001
	Sophomore (Year 2)	1489	33.1%	53.4%	13.5%		
	Junior (Year 3)	1515	36.7%	51.6%	11.7%		
	Senior (Year 4)	1284	34.3%	51.0%	14.7%		
Family Location	City/County	3206	32.2%	51.8%	16.0%	10.198	<0.05
	Town/Township	1547	34.5%	52.6%	12.9%		
	Village	1793	34.7%	50.7%	14.6%		
Annual Family Income	≤ 50,000 RMB	3840	32.5%	51.5%	16.0	23.602	<0.001
	50,000–100,000 RMB	1681	36.1%	51.8%	12.1%		
	100,000–150,000 RMB	670	33.3%	53.4%	13.3%		
	> 150,000 RMB	355	31.0%	49.9%	19.2%		
Only Child Status	Only child	3610	32.2%	51.0%	16.8%	23.493	<0.001
	Non-only child	2936	34.9%	52.6%	12.6%		

Note:N = 6,546.

An examination of differences in rural teaching willingness among pre-service teachers from local universities in terms of each dimension and the overall score based on their professional competence development levels revealed that all differences were statistically significant.Furthermore, the effect size η^2^ was relatively large, suggesting that the level of professional competence development exerts a substantial influence on rural teaching willingness among pre-service teachers from local universities. Post-hoc tests revealed significant pairwise score differences among the three groups of these pre-service teachers. The scores for overall willingness to teach in rural areas and the three sub-items, ranked from highest to lowest, were: the Excellence-Prepared Profile > the Development-Oriented Profile > the Weak Foundation Profile.

An analysis of demographic variables including gender, year of study, place of family residence, annual household income, and whether the student is an only child revealed differences in overall scores and individual item responses regarding rural teaching preferences among the three categories of teacher training students. The results indicate: (1) Third-year “foundation-weak” teacher training students demonstrated significantly lower willingness to teach in rural schools compared to the other two groups of the same academic year; (2) Among “foundation-weak” teacher trainees with annual household incomes of RMB 100,000–150,000, their willingness to integrate into rural environments, teach at rural schools, and interact with rural teachers was significantly lower than that of the other two groups. For those with annual household incomes exceeding RMB 150,000, the disadvantage in their willingness to interact with rural teachers was particularly pronounced.

Addressing these phenomena, the third year of study represents a pivotal transition period for teacher training students’ career orientation, during which their career decisions exhibit certain contradictory characteristics. At this stage, students’ anxiety regarding career-environment alignment increases significantly [32], teacher trainees with weaker foundational skills, owing to insufficient practical teaching abilities and an as-yet-unestablished commitment to rural teaching, are prone to a diminished willingness to engage when confronted with the distinctive characteristics of rural education posts—namely small class sizes, interdisciplinary teaching, and a strong emphasis on practical application. This stems from their comparatively low sense of self-efficacy, in stark contrast to the higher levels of professional competence confidence and value recognition exhibited by the other two cohorts in the same year group.

Secondly, according to cultural capital theory [33], households with annual incomes between RMB 100,000 and RMB 150,000 exhibit the characteristics of “suspended cultural capital”. Their aspirations for upward occupational mobility clash with the grassroots orientation of rural education, which reinforces students’ anxiety about downward mobility through familial cultural transmission. Familial emotional support fails to motivate students to commit to rural communities, resulting in a markedly lower willingness to integrate into rural environments. Conversely, households with annual incomes exceeding RMB 150,000 possess social networks and cultural capital that are more aligned with urban spheres. Students from such backgrounds face cultural and cognitive barriers to social integration with rural teaching communities.

This aligns with the findings of Pang et al. [34], who observed that “students from high-income families exhibit a significantly lower willingness to teach in rural areas compared to those from low-income families.”

### 3.4. Further study: Multiple linear regression analysis

This study adopts the classical theoretical model proposed by Hanushek to explore factors affecting rural teaching willingness among local university pre-service teachers across different professional competence profiles:


$$\begin{array}{c} Y=F(D,F,U,P) \end{array}$$ (1)

Here, Y denotes willingness to teach in rural areas, D represents demographic control variables, F signifies family factors, U indicates university factors, and P stands for policy factors. Multiple linear regression is a mathematical method used to construct regression equations involving two or more independent variables, thereby identifying linear relationships between these independent variables and the dependent variable. Its basic form is:


$$\begin{array}{c} y=\beta_{\text{0}}+\beta_{\text{1}}X_{\text{1}}+\beta_{\text{2}}X_{\text{2}}+\ldots +\beta_{p}X_{p}+\epsilon \end{array}$$ (2)

Among these, β_1_…β_p_ are termed regression coefficients, β_0_ is the constant term, ε represents the random error term, and p denotes the number of independent variables. The model is solved using the ordinary least squares (OLS) method, with the residual sum of squares function Q defined as:


$$\begin{array}{c} Q(\beta_{\text{0}},\beta_{\text{1}},\ldots ,\beta_{\text{20}})=\sum_{i=\text{1}}^{n}(y_{i}-\beta_{\text{0}}-\beta_{\text{1}}X_{i\text{1}}-\beta_{\text{2}}X_{i\text{2}}-\ldots -\beta_{\text{20}}X_{i\text{20}}{)}^{\text{2}} \end{array}$$ (3)

To minimise Q, we take the partial derivatives with respect to β_j (j = 0, 1,..., 20) and set them equal to zero, yielding the following system of normal equations:


$$\begin{array}{c} \left\{ \begin{array}{cc} \mathrm{-20pt}\frac{\partial Q}{\partial \beta_{\text{0}}}=-\text{2}\sum_{i=\text{1}}^{n}(y_{i}-\beta_{\text{0}}-\beta_{\text{1}}X_{i\text{1}}-\ldots -\beta_{\text{20}}X_{i\text{20}})=\text{0} & \\ \mathrm{-8pt}\frac{\partial Q}{\partial \beta_{\text{1}}}=-\text{2}\sum_{i=\text{1}}^{n}(y_{i}-\beta_{\text{0}}-\beta_{\text{1}}X_{i\text{1}}-\ldots -\beta_{\text{20}}X_{i\text{20}})X_{i\text{1}}=\text{0} & \\ \vdots & \end{array}\right. \end{array}$$ (4)

Solving the aforementioned system of normalized equations yields marginal effect estimates for each factor influencing willingness to teach in rural areas. This study constructed four models (Table 4) using multiple linear regression analysis to examine the determinants of rural teaching willingness across three groups of pre-service teachers from local universities, categorized by their professional competence profiles. Specifically, Model II adds the “family support” factor to Model I; Model III incorporates the “university teacher training objectives” factor into Model II; and Model IV introduces the “policy orientation” factor into Model III. In all models, the p-value of the F-statistic is less than 0.01, which significantly rejects the null hypothesis that all regression coefficients are zero. The coefficients of determination (i.e., explanatory power) R^2^ for Model IV across the three groups of pre-service teachers are 0.189, 0.280, and 0.401, respectively, which are significantly greater than those of Models I, II, and III. This indicates that the coefficients of determination of the models are statistically significant.

**Table 4:** Multiple linear regression analysis.

The Weak Foundation Profile					The Development-Oriented Profile				The Excellence-Prepared Profile			
Predictor Variables	Model I	Model II	Model III	Model IV	Model I	Model II	Model III	Model IV	Model I	Model II	Model III	Model IV
Constant	—	—	—	—	—	—	—	—	—	—	—	—
Gender	−0.011	−0.036	−0.030	−0.030	−0.063***	−0.030*	−0.021	−0.022	−0.118***	−0.070*	−0.051	−0.034
Grade	−0.037	−0.027	−0.024	−0.024	−0.008	−0.001	0.012	0.006	0.008	−0.006	0.000	−0.027
Family location	0.032	0.012	0.010	0.010	0.072***	0.027	0.034*	0.036*	0.092**	0.094**	0.097**	0.101***
Only child status	0.067**	0.065**	0.062**	0.062**	0.049**	0.039*	0.038*	0.041**	0.015	−0.004	−0.003	−0.003
Annual family income	−0.073**	−0.049*	−0.045*	−0.045*	−0.040*	−0.004	0.012	0.013	−0.071*	−0.033	−0.012	−0.002
A1		0.034	0.029	0.030		0.035*	0.016	0.005		0.086**	0.064*	0.003
A2		0.169***	0.171***	0.170***		0.108***	0.108***	0.111***		0.105***	0.099***	0.096***
A3		0.330***	0.313***	0.313***		0.463***	0.429***	0.414***		0.474***	0.410***	0.366***
B1			0.078***	0.079**			0.025	0.009			0.065	0.009
B2			−0.024	−0.025			−0.026	−0.043*			−0.029	−0.081
B3			0.077**	0.079**			0.055*	0.056*			0.103*	0.109*
B4			−0.087**	−0.083**			−0.001	−0.011			0.028	0.029
B5			0.052	0.050			0.074**	0.060*			0.019	−0.027
C1				0.011				0.066**				0.092*
C2				−0.035				−0.043				−0.042
C3				−0.007				−0.064				−0.053
C4				0.023				0.047				0.246**
C5				0.013				0.009				−0.032
C6				0.036				0.022				0.075
C7				−0.047				0.071*				0.018
R^2^	0.124	0.175	0.188	0.189	0.017	0.260	0.271	0.280	0.033	0.331	0.351	0.401
Adjusted R^2^	0.013	0.172	0.183	0.181	0.016	0.258	0.268	0.275	0.028	0.325	0.342	0.389
ΔR²	0.015	0.160	0.012	0.001	0.017	0.243	0.011	0.009	0.033	0.297	0.020	0.050
F value	6.849***	140.627***	6.647***	0.437	11.754***	368.448***	10.436***	5.749***	6.712***	142.943***	6.049***	11.406***

*Note:*

A1–A3 represent family economic situation, parental expectations, and parental attitudes;

B1–B5 represent regional talent cultivation positioning, localized curriculum system construction, contextualized practical teaching design, regionally embedded campus cultural education, and rural-oriented employment guidance mechanism;

C1–C7 represent special post policy guidance, salary and benefits improvement, preferential treatment in professional title evaluation and appointment, support for capacity building, incentives for professional identity, improvement of living conditions, and student loan compensation.

*p <0.05; **p <0.01;***p <0.001; N =6,546.

For teacher education students in the Weak Foundation Profile, Model I (containing only demographic variables) explains 1.5% of the variance in willingness to teach in rural areas. After incorporating family-related variables into Model II, the explanatory power surges to 17.5% (ΔR^2^ = 0.160), indicating that family support variables represent the core predictive factors in this sample. Among these family-related variables, parental attitudes (β = 0.313, p < 0.001) and parental expectations (β = 0.170, p < 0.001) exert significant positive effects, whereas family economic status shows a significant negative coefficient (β = −0.045, p < 0.05). That is, higher family economic affluence is associated with lower rural teaching willingness among these teacher education students.

Following the introduction of university-level factors in Model III, the cumulative explanatory power increased to 18.8%, with an incremental contribution of only 1.2%. Both regional talent cultivation positioning (β = 0.078, p < 0.05) and contextualized practical teaching design (β = 0.077, p < 0.05) exert significant positive effects. Conversely, regionally embedded campus cultural education shows a significant negative effect (β = −0.087, p < 0.05), indicating that inadequate guidance from universities on the value of rural education weakens these students’ willingness to pursue rural teaching posts.

Model IV, which incorporates policy factors, shows virtually no change in R^2^, and all policy variables are statistically insignificant. This indicates that policy dividends remain largely unrecognized by this cohort of teacher education students. The final model achieves an overall explanatory power of 18.9%, with family support accounting for the primary incremental increase. The positioning of university teacher training objectives makes a partial contribution, while the driving effect of policy orientation remains inconclusive.

For pre-service teachers at “Development-Oriented Profile” local universities, Model I explains merely 1.7% of the variance in rural teaching willingness. After incorporating family-level variables into Model II, the explanatory power surges to 26.0% (ΔR^2^ = 0.243, p < 0.001), accounting for 87% of the total variance in the final model. Among these variables, parental attitudes (β = 0.463, p < 0.001) exert the strongest effect, followed by parental expectations (β = 0.108, p < 0.001), both of which constitute significant positive drivers. Compared with students with weaker academic foundations, family economic status (β = 0.035, p < 0.05) shows a significant positive effect. This further indicates that when families are not constrained by financial pressures, economic resources improve the feasibility for these pre-service teachers to choose rural teaching positions.

After incorporating university factors into Model III, R^2^ increases to 27.1%, with an incremental explanatory power of 1.1%. In Model IV, which includes policy factors, R^2^ rises to 28.0%, representing an incremental increase of 0.9%. Within this model, special post policy guidance (β = 0.066, p < 0.01) and compensatory student loans (β = 0.071, p < 0.05) show significant positive effects, indicating that this cohort is sensitive to the dual advantages of lower entry thresholds and debt relief associated with rural special post teacher programs.

The final model yields an explanatory power of 28.0%, in which family support provides the primary incremental contribution, university teacher training objectives make a relatively weaker contribution, and policy orientation plays a partial auxiliary role.

Pre-service teachers from local universities in the Excellence-Prepared Profile show higher initial willingness to engage in rural teaching (with R^2^ = 3.3% in the Model I). Subsequently, after incorporating family support variables into the Model II, the explanatory power significantly and substantially increases by 29.7% to 33.1%, and specifically, the parental attitudes (β = 0.474, p < 0.001) and the parental expectations (β = 0.105, p < 0.001) consistently maintain significant positive effects.

The Model III, which incorporates university teacher training objectives, elevates the explanatory power to 35.1%. Specifically, contextualized practical teaching design yields a significantly positive effect, indicating that local university teacher education students emphasize integrating coursework with local characteristics. The Model IV, which introduces policy orientation factors, significantly increases R^2^ to 40.1%—i.e., the highest explanatory power among the three models. Specifically, both the special post policy guidance (β = 0.092, p < 0.05) and the capability enhancement support (β = 0.246, p < 0.01) exert significant positive effects, which further indicates that outstanding local university teacher education students value the expanded rural teaching career pathways brought by policy dividends and the improvement of future professional competence, both of which exert considerable appeal.

The final model achieves an overall explanatory power of 40.1% for the dependent variable, and family support remains the dominant predictive factor. The reasonable positioning of university teacher training objectives within this model framework produces a so-called “adding icing on the cake” effect and further significantly unleashes policy dividends.

### 3.5. Multi-group mediation effect test

Structural equation modelling was applied to examine the underlying mechanisms linking family support, university teacher training objectives, and the professional competence development of local university teacher education students to their willingness to teach in rural areas. The full-sample model exhibits goodness-of-fit indices: χ^2^/df = 6.083, CFI = 0.970, TLI = 0.913, IFI = 0.941, RMSEA = 0.075, which are all within standard academic reference ranges, indicating that the model has excellent fit.

Path analysis results reveal that family support and university teacher training objectives exert significant positive effects on the professional competence development of local university teacher education students, while their professional competence, in turn, significantly and positively predicts their willingness to teach in rural areas. Furthermore, the Bootstrap mediation analysis method specifically reveals that the 95% confidence intervals for the mediating effects of professional competence development on rural teaching willingness are [0.004, 0.041] for family support, [0.002, 0.052] for university teacher training objectives, and [−0.006, −0.001] for policy orientation, with all intervals excluding zero, which indicates that family support, university teacher training objectives, and policy orientation not only directly predict rural teaching willingness among local university teacher education students but also indirectly influence it through their professional competence development.

To specifically examine whether the predictive effects of family support, university teacher training objectives, and professional competence on willingness to teach in rural areas among local university teacher education students differ across distinct local university teacher education student cohorts, this study adopted multi-group structural equation modelling analysis for comparative analysis (Table 5), and used the three identified profiles—the Weak Foundation Profile, the Development-Oriented Profile, and the Excellence-Prepared Profile—as the grouping variables. The results indicate that p-values for comparisons between the measurement Weights model, structural Weight model, structural Covariance model, structural Residuals model, and measurement Residuals model were all below 0.001, signifying significant differences in path coefficients across the three student cohort models. In addition, the overall model fit indices indicate satisfactory overall model fit: namely χ^2^/df ranged from 9.452 to 10.019, NFI from 0.902 to 0.940, IFI from 0.903 to 0.913, CFI from 0.901 to 0.904, and RMSEA from 0.041 to 0.049.

**Table 5:** Multi-group mediation effects.

Path	Total Sample		The Weak Foundation Profile		The Development-Oriented Profile		The Excellence-Prepared Profile	
	*β*	*SE*	*β*	*SE*	*β*	*SE*	*β*	*SE*
Family support → Rural teaching willingness	0.651***	0.049	0.450***	0.045	0.559***	0.057	0.526***	0.037
University teacher training objectives → Rural teaching willingness	−0.101***	0.018	−0.053***	0.013	−0.060***	0.014	−0.112***	0.021
Policy orientation → Rural teaching willingness	−0.066***	0.014	−0.096***	0.016	−0.048***	0.011	−0.032*	0.017
Family support → Professional competence	0.275***	0.026	0.641***	0.059	0.541***	0.043	0.437***	0.037
University teacher training objectives → Professional competence	0.288***	0.015	0.107***	0.031	0.162***	0.024	0.292***	0.021
Policy orientation → Professional competence	−0.022*	0.011	0.057*	0.026	0.066***	0.015	0.049**	0.017
Professional competence → Rural teaching willingness	0.095**	0.032	0.141**	0.045	0.004	0.052	0.126**	0.043
Family support → Professional competence → Rural teaching willingness	0.026*	0.009	0.099*	0.036	0.030	0.023	0.055*	0.023
University teacher training objectives → Professional competence → Rural teaching willingness	0.027*	0.013	0.015*	0.010	0.048	0.036	0.037*	0.019
Policy orientation → Professional competence → Rural teaching willingness	−0.002*	0.001	0.008	0.006	0.007	0.006	0.006*	0.005

Note:*p < 0.05; **p < 0.01;***p < 0.001; N = 6,546.

For the Weak Foundation Profile, family support (β = 0.450, p < 0.001) positively predicted their willingness to teach in rural areas. Conversely, university teacher training objectives (β = −0.053, p < 0.001) and policy orientation (β = −0.096, p < 0.001) negatively predicted their willingness to teach in rural areas. Moreover, the professional competence of this category of local university teacher education students does not significantly mediate the relationship between policy orientation and their willingness to teach in rural areas. However, it instead partially mediated the relationship between family support, university teacher training objectives and their willingness to teach in rural areas, with mediation effect sizes of 0.099 (p < 0.05) and 0.015 (p < 0.05), accounting for 21.9% and 3.7% of the total effect respectively.

For the Development-Oriented Profile, family support (β = 0.559, p < 0.001) positively predicted their willingness to teach in rural areas. Conversely, university teacher training objectives (β = −0.060, p < 0.001) and policy orientation (β = −0.048, p < 0.001) negatively predicted their willingness to teach in rural areas. Moreover, the professional competence of this category of local university students does not significantly mediate the influence paths between family support, university teacher training objectives, policy orientation and their willingness to teach in rural areas. This indicates that the rural teaching willingness of the Development-Oriented Profile of pre-service teachers from local universities is primarily driven by family factors, including family values, financial support, and family responsibilities.

For the Excellence-Prepared Profile, family support (β = 0.526, p < 0.001) positively predicted their willingness to teach in rural areas. University teacher training objectives (β = −0.112, p < 0.001) and policy orientation (β = −0.032, p < 0.001) negatively predicted their willingness. Professional competence significantly mediated the influence of family support, university teacher training objectives, and policy orientation on rural teaching willingness. Professional competence significantly mediated all three pathways linking family support, university teacher training objectives, and policy orientation to rural teaching willingness, and with mediation effect sizes of 0.055 (p < 0.05), 0.037 (p < 0.05), and 0.006 (p < 0.05), accounting for 11.5%, 7.7%, and 1.3% of the total effect respectively. This indicates that the rural teaching willingness of the Excellence-Prepared Profile of pre-service teachers from local universities is directly positively influenced by family support, while directly negatively influenced by university teacher training objectives and policy orientation. In addition, these factors collectively act on their professional competence development, thereby indirectly influencing their rural teaching willingness.

## 4. Discussion and suggestion

### 4.1. Discussion

Using an individual-centreed latent profile analysis approach, this study reveals significant heterogeneity in the professional competence of pre-service teachers at local universities. Three distinct professional competence profiles emerge: the Weak Foundation Profile, the Development-Oriented Profile, and the Excellence-Prepared Profile. This indicates that single-variable research methods cannot fully capture the true state of professional competence among pre-service teachers at local universities, necessitating a categorized exploration of this group.

Teacher education students in the Weak Foundation Profile obtained the lowest across all five professional competency dimensions compared to the other two profiles: professional learning literacy (M = 3.09), practical teaching competency (M = 3.03), occupational post adaptability (M = 3.14), career development planning (M = 3.02), and rural educational perception (M = 3.06). While practical teaching competency is among the highest-scoring dimensions in the full sample, it ranks among the lowest within this subgroup, alongside career development planning. These findings reveal deficiencies in the essential competencies required for adapting to professional demands during teacher education, particularly in grassroots education settings.

Particularly concerning were practical teaching competency and career development planning, which ranked last among all dimensions. This reveals a widespread lack of essential professional competencies and weak commitment to serving grassroots education among teacher education students during their training.Zhou et al. specifically noted that a group of teacher education students exhibiting “hesitation” in their commitment to returning to their hometowns and fulfilling their obligations lack clear plans for work and life. They struggle to weigh or articulate the value and meaning of “returning home” for themselves, leading to indecision about teaching in rural areas [35].

Such local university teacher education students lack the pedagogical competence and professional conviction required to root themselves in rural teaching practice. In practice, they often struggle to effectively address challenges in rural school settings due to their skill gaps, value confusion, and unclear career development trajectories. Teaching in rural areas appears to them as a mere passive and reluctant choice, as they have not yet achieved a state of development where they are “suited to grassroots educational needs and equipped with solid professional capabilities”.

The other two categories of local university teacher education students—the Development-Oriented Profile and the Excellence-Prepared Profile—both scored lower on the “rural educational perception” dimension than on the other four sub-dimensions. The scores of teacher education students belonging to the Development-Oriented Profile were 3.81, 3.97, 3.74, 3.55, and 3.45 (with the “rural educational perception” dimension score being the lowest), while those of students in the Excellence-Prepared Profile were 4.72, 4.96, 4.29, 4.07, and 3.91 (with the “rural educational perception” dimension score being the lowest). Even local university teacher education students with solid professional competence and development potential demonstrated insufficient understanding of rural education, which indicates that blurred perceptions of rural schools and education are a common shortcoming in the professional competence cultivation of teacher education students.

This deficiency is fundamentally manifested as a lack of comprehensive and systematic understanding of rural school settings, rural educational needs, and the developmental potential of rural education [36]. However, understanding rural education is a crucial component of developing a connection to their homeland. If teacher education students fail to establish a scientific understanding of rural education, even though they have solid professional competence, they will struggle to develop a steadfast commitment to pursuing rural teaching posts.

For local university pre-service teachers in the Weak Foundation Profile, Development-Oriented Profile, and Excellence-Prepared Profile groups, rural teaching willingness is shaped by the interplay of multiple factors, including individual characteristics and environmental conditions. Significant group differences exist in the underlying mechanisms of these influences.

Firstly, the mediating role of family support pathways plays a dominant part in shaping the professional competence of teacher education students in the Weak Foundation Profile. Atkinson’s early model of Expectancy-Value Theory posits that “individual task selection motivation depends on the subjective perception of the difficulty of the task” [37]. Rural teachers are generally expected to be “specialized in one area yet versatile in others.” Weak Foundation Profile teacher education students exhibit deficiencies in practical teaching competency and occupational post adaptability. They perceive rural teaching as highly challenging yet low-rewarding, making it difficult for them to effectively integrate the role of rural teacher into their professional narratives and future plans. Consequently, their willingness to pursue rural teaching posts remains inadequately stimulated.

The mediation analysis incorporating professional competence revealed a significant mediating effect between environmental factors and rural teaching willingness, primarily driven by the “family support” pathway. The regression coefficient for family support on professional competence reached 0.641 (p < 0.001), while the coefficient for professional competence on rural teaching willingness was 0.141 (p < 0.01)—which were significantly higher than those in the other two teacher education groups. This indicates that material investment and emotional support from families can convert external resources into professional development capital for these Weak Foundation Profile teacher education students. In turn, this capital compensates for their initial disadvantages, strengthens the role of professional competence in supporting their decision to teach in rural areas, and consequently enhances their willingness to teach in rural areas.

Furthermore, the mediating effect of university teacher training objectives is equally significant and positive, indicating that the grassroots relevance of these objectives is crucial for bridging the gap between pre-service teachers’ professional competence development and their willingness to teach in rural areas. When training objectives explicitly aim to serve rural education, curriculum design and practical arrangements become more aligned with rural educational needs, thereby simultaneously promoting and enhancing pre-service teachers’ professional competence and their willingness to do so.

Unlike the previous two factors, policy orientation exerts the strongest negative effect on the rural teaching willingness of Weak Foundation Profile teacher education students (β = −0.096, p < 0.001), and this negative effect is not mitigated by the mediating effect of professional competence. This indicates that for these Weak Foundation Profile local university pre-service teachers, the communication and interpretation of rural teaching policies may fail to align with their current capabilities and psychological expectations. Instead, policy statements such as “grassroots service” and “guaranteed conditions” may exacerbate their anxiety about the mismatch of their capabilities for rural teaching, thus leading to resistance. This aligns with Table 4’s finding of overall policy ineffectiveness among this group.

Secondly, the formation of rural teaching intentions among teacher education students in the Development-Oriented Profile does not involve the mediating effect of professional competence. The professional competence of teacher education students in the Development-Oriented Profile falls within the mid-to-high range. While environmental factors such as family support, university teacher training objectives, and policy orientation significantly influence their professional competence, the direct effect of professional competence on their willingness to teach in rural areas does not reach statistical significance (β = 0.004, p > 0.05). The indirect effects across all three pathways were also insignificant, indicating that this group of students’ willingness to teach in rural areas does not depend on the mediating role of professional competence. Instead, external environmental factors exert a stronger influence on their rural teaching intentions. Notably, family support exerts a strong positive influence on this group’s willingness to teach in rural areas (β = 0.559, p < 0.001), signifying that family support is the core factor driving their willingness to teach in rural areas.

A possible reason lies in the hierarchical change in the career choice needs of this group, which is gradually transitioning from the basic need of “professional competence attainment” to the higher-order need of “value alignment”. When considering rural teaching, they focus on factors such as the developmental potential of rural education, compatibility with the regional lifestyle, and career development prospects of rural teaching [38], whether non-professional competencies are sufficient to support their job performance, and whether familial emotional attachments embedded in interpersonal relationship networks [39], the value support derived from regional affiliation aligns with the aforementioned higher-order needs, thus effectively enhancing these Development-Oriented Profile teacher education students’ level of psychological acceptance of teaching in rural areas.

At the same time, teacher education students in the Development-Oriented Profile are in the upward development stage of professional competence and are highly sensitive to policy signals including employment entry barriers and student debt relief. Consequently, policy guidance significantly promotes the accumulation of their professional competence (β = 0.066, p < 0.001). Nevertheless, policy guidance fails to elevate their willingness to teach in rural areas through professional competence (β = 0.007, p > 0.05). This indicates that factors such as relatively easy access to rural teaching positions after graduation and the benefits of tuition reductions act primarily as short-term incentives for enhancing their professional competence during their studies. However, these are insufficient to translate into core motivations sustaining their long-term commitment to rural teaching or to alter their long-term career planning.

This precisely reflects that a significant portion of rural teaching choices among local university teacher education students are driven more by the immediate benefits released by policy incentives than by the establishment of a deep connection between rural education development opportunities and their own professional value realization. Especially when they are faced with the diverse career options available in cities, such teacher education students in the Development-Oriented Profile with solid professional competence foundations may strive to enhance themselves based on their personal strengths, professional advantages, values, and needs. However, once their goals are achieved, they may not necessarily stick to a single career path but instead continually seek new professional challenges, and thus the allure of teaching in rural areas for them is diluted.

Thirdly, the decision of pre-service teachers in the Excellence-Prepared Profile to take up hometown rural teaching is driven by the combined effects of environmental factors and professional competence. The professional competence of pre-service teachers in the Excellence-Prepared Profile not only positively affects their rural teaching willingness, but also exerts a significant mediating effect on the influencing paths of family support, university teacher training objectives, and policy orientation toward such willingness. This further corroborates the perspective of The social cognitive career theory that “individual career intentions are the result of the interaction between external environments and individual factors”.

Notably, the mediating effect of professional competence within the pathway driven by university teacher training objectives reached 7.1% — which is the highest among the three categories of teacher education student groups. This indicates that when university teacher training incorporates local characteristics and emphasizes the regional specificity of practical teaching, it can be effectively perceived by teacher-trainees in the Excellence-Prepared Profile, thus aiding them in developing a clear understanding of rural teaching positions. In turn, this in-depth understanding further motivates teacher education students in the Excellence-Prepared Profile to actively improve their professional learning capabilities, practical teaching competency and occupational post adaptability, thereby indirectly boosting their willingness to engage in rural teaching.

The mediating effect of the policy orientation pathway is 0.006 (p < 0.05), indicating that policy dividends delivered via initiatives including the Special Post Teacher Scheme and the Rural Teacher Capacity Enhancement Support Programme can be fully perceived by teacher education students in the Excellence-Prepared Profile. Meanwhile, high professional competence, acting as a conduit for policy dividends, further amplifies the promotional role of these policy dividends in fostering their willingness to teach in rural areas. In this process, policy support ceases to be an externally imposed condition and instead transforms into an endogenous resource that these teacher education students can proactively utilize and leverage, effectively safeguarding their sense of professional fulfillment in their development in rural teaching.

From an individual perspective, pre-service teachers in the Excellence-Prepared Profile have gradually shifted their cognitive orientation toward hometown rural teaching from a “capability-driven” orientation to a “willingness-driven” one. In this process, professional competence serves as a vital bridge connecting their reality with their aspirations, thus facilitating the transformation of these pre-service teachers’ specialised yet versatile professional skills into a deep-rooted long-term educational commitment to rural communities [40], their professional competence serves as a crucial anchor for consolidating their commitment to teaching at the grassroots level. Yet it must also be recognized that teacher education students in the Excellence-Prepared Profile account for the smallest proportion among the three profiles, which reflects the limited effectiveness of local universities in cultivating outstanding reserve talents for rural teaching. There remains a gap between the supply of high-calibre, well-suited and highly committed rural teachers and the demands of high-quality development in rural education.

Furthermore, a comparative analysis of the mechanisms influencing rural teaching willingness among three categories of teacher education students revealed two key findings: Firstly, the impact of policy orientation on rural teaching willingness among local university teacher education students exhibited a “gradient decrease” feature. Within the full sample model, policy orientation had a negative impact on rural teaching willingness, with a coefficient of (β = −0.066, p < 0.001). After grouping the samples, this coefficient exhibited a monotonically decreasing trend.

Compared with teacher education students with low professional competence who tend to develop psychological resistance to policy orientation, those with high professional competence can utilize policy orientation as a reference point for their career choice of teaching in rural areas. This indirectly reflects that the degree of alignment between policy orientation and individual professional competence is more critical than policy intensity itself. Instead, this fundamentally represents a coupling process between external policy drivers and internal individual recognition. Only when policy supply aligns closely with teacher education students’ career development planning can the resistance from individuals during policy implementation be genuinely mitigated.

The direct effect of professional competence on willingness to teach in rural areas exhibits a U-shaped distribution across the three categories of teacher education students, with high values at both ends and a low value in the middle of the distribution. For teacher education students in the Weak Foundation Profile, professional competence acts as a bargaining chip to avoid downward occupational mobility, thus yielding the strongest influence on their willingness to teach in rural areas (β = 0.141, p < 0.01).

For teacher education students in the Excellence-Prepared Profile, professional competence acts as a vehicle for realizing their rural educational ideals and personal value, producing the second-strongest effect (β = 0.126, p < 0.01). For teacher education students in the Development-Oriented Profile who lie in the middle range of professional competence, their concerns about regional preferences and career advancement opportunities mean that improvements in professional competence have limited influence on their decision to return to their hometowns to teach, thus resulting in the weakest effect (β = 0.004, p > 0.05). This also reflects that when making career choices, the role of professional competence among local university teacher education students becomes more pronounced in either mitigating downward risks or realizing upward value.

### 4.2. Suggestion

Pre-service teachers from local universities exhibit significant group heterogeneity in their professional competence development, and their willingness to teach in rural areas also differs fundamentally due to the combined influences of individual and environmental factors. It is necessary to adhere to the principle of targeted classification policies, integrate the joint forces of family support, university training, and policy guarantee, and promote a benign two-way interaction between the improvement of professional competence and the enhancement of rural teaching willingness among pre-service teachers in local universities.

First, for the Weak Foundation Profile: Strengthen the enabling role of family support through policy guidance. The core dilemmas of the Weak Foundation Profile pre-service teachers are insufficient professional competence and cognitive biases toward rural teaching. Their willingness to teach in rural areas is particularly weak among those from middle and high-income families. Accordingly, amplifying the enabling effect of family support through policy guidance is the key to resolving such dilemmas. Student counselors, class supervisors, and other frontline educators should actively serve as a connecting link, organizing targeted policy briefings on rural education and experience-sharing sessions with outstanding alumni who teach in rural areas, so as to provide practical references for pre-service teachers to accurately understand rural education and establish teaching commitment. On the one hand, provincial and higher-level supporting policies should be systematically interpreted, including preferential points in professional title evaluation after three years of rural service, opportunities for exchange and rotation in high-quality urban schools within five years, as well as policy benefits such as housing security and living allowances, to strengthen the value guidance of policy orientation.

On the other hand, outstanding alumni who have rooted themselves in rural schools for 2–3 years and achieved initial professional advancement should be invited to share their growth paths from “campus classroom” to “rural context”, enabling students to fully perceive the career sustainability of rural teaching, correcting misconceptions such as viewing rural teaching as a temporary transition with no developmental prospects, and helping such pre-service teachers deepen their understanding of the social value and career potential of rural education. In this way, family emotional support can be effectively transformed into endogenous motivation for improving professional competence and alleviating anxiety about career “downward mobility”. In addition, focusing on the junior year as a critical period for career orientation, an innovative dual-mentor model can be implemented, combining university instructors and rural key teachers. Microteaching training can be provided to address deficiencies in practical teaching skills, and teaching practice can be extended to real rural classrooms through field studies in rural schools. Meanwhile, immersive activities such as shadowing in rural teacher communities and academic support for left-behind children can be designed to help students adapt to rural education settings and collaborative relationships, remedy shortcomings in professional competence, and achieve synchronous progress in competence development and rural education cognition.

Secondly, for the Development-Oriented Profile: Optimize localized training and incentive pathways. The professional competence of Developmenl-Oriented Profile pre-service teachers has surpassed the basic level of competency, and their career choices have gradually shifted from capability matching to the pursuit of personal value realization. Therefore, intervention strategies should go beyond the traditional focus on merely improving professional competence and align with their regional preferences and upward development needs.Local universities should optimize their curriculum systems by offering courses on rural revitalization, left-behind children education, and the development of rural characteristic educational resources. Localized modules such as frontier rural education policies and practices, rural curriculum development, and generalist teaching strategies in small rural schools should be added. Pre-service teachers should be guided to conduct research based on real rural school scenarios to achieve precise integration of theoretical learning and grassroots educational needs.

Local governments should strengthen long-term incentives in policy supply, linking performance rewards with professional development resources—such as priority access to mentoring by county-level master teachers at the initial stage of employment, priority recommendation for provincial and municipal research projects in the medium term, and long-term training opportunities in high-quality urban schools with inclusion in the county-level reserve talent pool—to form a clear upward development path. Meanwhile, a comprehensive security plan for local talent return should be established, supporting region-specific policies such as transitional housing and priority enrollment for children, so that pre-service teachers’ regional identity and developmental aspirations can be transformed into a sustained “pull force” for rural service, rather than short-term motivation driven solely by policy benefits.

Finally, for the Excellence-Prepared Profile: Build a growth system for rural education leaders. A practical gap remains in transforming individually outstanding Excellence-Prepared Profile pre-service teachers into a scalable reserve of rural teachers. It is necessary to strengthen coordination between external support systems and individual career development, and expand the cultivation of potential rural education leaders through institutional innovation.Based on the view in Social Cognitive Career Theory (SCCT) that environment and individual factors jointly shape career inclination, universities and local education authorities should establish a linkage mechanism between elite pre-service teachers and high-quality rural schools. For example, a “Shadow Principal” program can be launched in the final academic year, allowing high-ability pre-service teachers with rural teaching enthusiasm to participate in grassroots school management practices, fostering their courage to “go to rural areas”, sense of identity to “stay grounded”, and professional confidence to “teach well”. A clear three-stage growth channel for rural education leaders can be designed: those with excellent performance after one year of service qualify for provincial rural education training; those after two years may lead key rural school research projects; and those with outstanding performance after three years are included in the county-level reserve cadre system.

Furthermore, in the digital era, a digital-physical integrated rural education practice centre can be constructed, providing tailored support such as classroom problem diagnosis and rural curriculum development through online virtual simulation and offline school-based guidance. An electronic growth portfolio can also be established to dynamically track the full-cycle career development of pre-service teachers from university training and internship practice to in-service growth, providing a solid basis for individual constructing professional development profiles.

## Supporting information

S1 Data (XLS)

## References

[1] Weifen D. Hybrid training paths for rural teachers: Based on the concept of “the third space”. Educational Research. 2022;43(08):31–41.

[2] Shangfeng SU, Huang L. Guided reflow: The functional logic of the evolution of local government-funded pre-service teacher education policies—An analysis based on policy texts of 30 provinces. Educational Research. 2021;42(12):131–41.

[3] Shan C, M K, Guo L. Low-Quality Compliance and High-Quality Escape: Analysis of Factors Influencing the Return Intentions of Local Government-Oriented Pre-Service Teachers. Fudan Education Forum. 2025;23(01):82–91. doi: 10.13397/j.cnki.fef.2025.01.007

[4] Fang G. Research on the “local” value orientation of the cultivation of government-funded pre-service teachers. Teacher Education Research. 2022;34(04):39–44.

[5] Zhi Z. Research on the influencing factors of rural educational beliefs of government-funded oriented pre-service teachers. Hunan Normal University. 2022. doi: 10.27137/d.cnki.ghusu.2022.003196

[6] Feng Z, Yu J, Du Y. Research on the professional identity and its influencing factors of tuition-free pre-service teachers. Journal of Hebei Normal University (Educational Science Edition). 2010;12(07):69–75. doi: 10.13763/j.cnki.jhebnu.ese.2010.07.015

[7] Hao W, Rui Z. Analysis of influencing factors of employment intention of college students in northern Jiangsu returning to their hometowns based on push-pull theory. Heilongjiang Science. 2024;15(01):52–4.

[8] Guo L. Research on the mechanism of promoting normal graduates’ employment in rural primary and secondary schools. Teacher Education Research. 2011;23(06):19–24.

[9] Wenjin KE, Fei D, Jiang J. Analysis of influencing factors of rural teachers’ teaching intentions among normal students of rural origin: Based on a survey of normal universities in western China. Fudan Education Forum. 2018;16(04):73–9. doi: 10.13397/j.cnki.fef.2018.04.012

[10] Jiang J, Chen X. Analysis of normal students’ willingness to teach in rural areas and its influencing factors under the background of “rural teacher living allowance policy”: Based on a survey of 15 colleges and universities in impoverished areas of western China. Teacher Education Research. 2019;31(01):43–50. doi: 10.13445/j.cnki.t.e.r.2019.01.008

[11] Hongyu MA, Cai Y, Tang H. The internal structure and characteristics of pre-service teachers’ professional identity. Teacher Education Research. 2013;25(01):49–54.

[12] Jing Z. Research on the influencing factors and promotion strategies of normal students’ willingness to teach in rural areas. Harbin Normal University. 2022. doi: 10.27064/d.cnki.ghasu.2022.000306

[13] Yi X. Influencing factors and countermeasures of normal graduates’ teaching in local normal universities: Based on a survey in Hunan province. Journal of Hunan University of Humanities, Science and Technology. 2022;37(02):120–5.

[14] Deng S, Qiao L, Yue X. A follow-up survey on the teacher competency of government-funded oriented pre-service teachers: A case study of Hunan First Normal University. Journal of Inner Mongolia Normal University (Educational Science Edition). 2015;28(11):17–9.

[15] Watt HMG, Richardson PW. Motivational Factors Influencing Teaching as a Career Choice: Development and Validation of the FIT-Choice Scale. The Journal of Experimental Education. 2007;75(3):167–202. doi: 10.3200/jexe.75.3.167-202

[16] Liyan GUO, Bing W, Juan X, et al. Research on the Professional Development and Living Conditions of Newly Graduated Normal Students. Modern Education Management. 2017:(05): 88–92. doi: 10.16697/j.cnki.xdjygl.2017.05.015

[17] Xie Q, Rong J, Huang L. Government-funded pre-service teachers’ retention in rural areas from the perspective of relationships: Practical dilemmas and breakthrough paths—Based on in-depth interviews with pre-service teachers at School S. Teacher Education Research. 2022;34(04):86–92. doi: 10.13445/j.cnki.t.e.r.2022.04.004

[18] Wu C. Research on the Identity of Rural Novice Teachers. Sichuan Normal University. 2017.

[19] Guo F. Classification of career orientations of local government-funded pre-service teachers and their influencing factors. Journal of University of Jinan (Social Science Edition). 2024;34(02):150–8. doi: 10.20004/j.cnki.ujn.2024.02.014

[20] Wei C, Zhang X, Zhao H. Research on the types of professional identity and the characteristics of learning motivation of tuition-free pre-service teachers. Teacher Education Research. 2013;25(03):66–71.

[21] An X, Cao X. Who is more willing to stay in rural schools? An empirical analysis based on rural teachers’ mobility intentions. Journal of Educational Science of Hunan Normal University. 2017;16(04):12–5. doi: 10.19503/j.cnki.1671-6124.2017.04.003

[22] Zhu Y, Wang Y, Wu D. Multidimensional analysis and countermeasure examination of rural teaching intentions of local government-funded oriented pre-service teachers from a typological perspective. University Education Science. 2022;(01):64–71.

[23] Yu L. What competencies do pre-service teachers need most? From the perspective of three professional standards. Journal of Henan Institute of Science and Technology. 2019;39(04):52–5.

[24] Mei W, Tainian X, Pei X. Reconceptualizing learning: learners, contexts, and cultures—new progress in learning sciences research from “how people learn ii”. Open Education Research. 2019;25(06):46–57. doi: 10.13966/j.cnki.kfjyyj.2019.06.005

[25] Lent RW, Brown SD, Hackett G. Toward a unifying social-cognitive theory of career and academic interest, choice, and performance. Journal of Vocational Behavior. 1994;45(01):79–122. doi: 10.1006/jvbe.1994.1027

[26] Miller HA, Turner K, Henderson CE. Psychopathology of sex offenders: A comparison of males and females using latent profile analysis. Criminal Justice and Behavior. 2009;36(8):778–92. doi: 10.1177/0093854809336400

[27] Man W. Research on the status quo and influencing factors of professional identity of novice primary school teachers with non-normal majors. Northeast Normal University. 2024.

[28] Hui G. Discussion on strategies for improving professional quality and employability of students in normal universities. China Training. 2021;(03):74–5. doi: 10.14149/j.cnki.ct.2021.03.037

[29] Zhao H, Zhang X. The Impact of Educational Policies on the Teaching Motivation and Professional Identity of Tuition-free Pre-service Teachers. Journal of Beijing Normal University (Social Sciences). 2015;:51–9.

[30] Zhang X, Yao M, Li Q. The impact of policy satisfaction and teacher support on the occupational self-efficacy and professional identity of government-funded pre-service teachers: A longitudinal study. Journal of Beijing Normal University (Social Sciences). 2022;(06):50–9.

[31] Wen Z, Xie J, Wang H. Principles, steps, and procedures of latent class models. Journal of East China Normal University (Educational Sciences). 2023;41(01):1–15. doi: 10.16382/j.cnki.1000-5560.2023.01.001

[32] Huang S. Report on the Development of Rural Education in China. Beijing: Education Science Press. 2021. https://www.pishu.com.cn/skwx_ps/bookdetail?SiteID=14&ID=13444183

[33] Xiao R, Qiu L. Cultural Capital and Class Identity. Journal of the Chinese Academy of Governance. 2016;(06):59–64. doi: 10.14063/j.cnki.1008-9314.2016.06.008

[34] Chunmin P, Zhenmin T. Analysis of the impact of public service motivation on pre-service teachers’ intentions to teach in rural areas. Higher Education Exploration. 2023;(04):46–52.

[35] Dan Z, Kang M, Shan C. Typological analysis and enhancement strategies of rural-oriented pre-service teachers’ intentions to return home: Based on a survey of 2,514 students from 8 provinces. Journal of Soochow University (Educational Science Edition). 2024;12(03):95–107. doi: 10.19563/j.cnki.sdjk.2024.03.010

[36] Yujun G, Tao X. Pre-service teachers moving towards high-quality construction of rural teacher teams: Feasibility, difficulty, and necessity. Contemporary Teacher Education. 2024;17(01):82–9. doi: 10.16222/j.cnki.cte.2024.01.001

[37] Wigfield A, Eccles JS. The development of achievement task values: A theoretical analysis. Developmental Review. 1992;12(3):265–310. doi: 10.1016/0273-2297(92)90011-p

[38] Rong J, Xin L, Huang Y. Investigation on the rural primary school teaching intentions of government-funded pre-service teachers in local normal universities. Educational Research and Experiment. 2019;(06):29–34.

[39] Lei W, Zihan W. Why do excellent teachers root in rural areas? A perspective based on emotional geography. Research in Educational Development. 2023;43(18):1–8. doi: 10.14121/j.cnki.1008-3855.2023.18.002

[40] Sun X, Xi L, Hou Z. Research on the Impact of Professional Identity of Rural-oriented Pre-service Teachers on Their Intentions to Teach in Rural Areas. Journal of Educational Development. 2024;(06):87–96. doi: 10.16215/j.cnki.cn44-1371/g4.2024.06.006

